# IReCAPTCHA: a robust image-reasoning CAPTCHA system

**DOI:** 10.1186/s42400-025-00484-0

**Published:** 2026-04-13

**Authors:** Bidyut Das, Dilip K. Prasad, Arif Ahmed Sekh

**Affiliations:** 1 Department of Information Technology, Haldia Institute of Technology, Haldia, 721657 West Bengal India; 2 Department of Computer Science, UiT The Arctic University of Norway, Tromsø, 9017 Tromsø Norway

**Keywords:** Image-reasoning CAPTCHA, AI-resistant authentication, Deep learning attacks, Human verification systems, Automated bot detection, Web security defenses

## Abstract

CAPTCHAs are widely used as authentication methods in mobile applications and web-based services to prevent AI bots from gaining unauthorized access. Early CAPTCHAs relied on image-based techniques, distorting text within images to make it difficult for bots to decipher. However, modern AI-powered optical character recognition (OCR) systems can now easily bypass these measures. To address this limitation, image-reasoning CAPTCHAs were introduced. Basic versions of these rely solely on object detection, rendering them vulnerable to advanced AI attacks. In response, we propose a novel image-reasoning CAPTCHA, called IReCAPTCHA, which integrates image understanding, noise mitigation, and mathematical reasoning. Unlike traditional object detection-based CAPTCHAs, our approach challenges users with tasks that require the comprehension of multiple objects, counting, and color recognition. Additionally, we apply noise mitigation techniques to obscure visual information, thereby enhancing resistance to AI-based deciphering. To evaluate the effectiveness of our CAPTCHA, we developed a comprehensive dataset specifically designed to test its robustness against AI attacks. The results are highly promising, demonstrating significantly greater resistance compared to existing image-reasoning CAPTCHAs. These findings suggest that our approach has strong potential to enhance online security by effectively deterring automated access attempts by malicious bots.

## Introduction

The term CAPTCHA stands for “Completely Automated Public Turing Test to Tell Computers and Humans Apart" Searles (2024). In 2003, Von Ahn et al. (2003) developed the first CAPTCHA system. Since its inception, CAPTCHA technology has been applied across various domains, including network security, natural language processing, signal processing, and computer vision Hasan (2016). CAPTCHAs are widely used in commercial applications to safeguard against malicious programs and bots Bodkhe et al. (2017). The principle behind CAPTCHAs is to create challenges that are difficult for bots to solve but relatively easy for humans. An effective CAPTCHA should achieve a human success rate of 90% or higher while ensuring a success rate of less than 1% for computer programs Bursztein et al. (2011).

Various CAPTCHA methods have been proposed over the years, typically involving recognition tasks presented to users. Among the earliest are text-based CAPTCHAs (Wang et al 2023a) and audio-based CAPTCHAs (Sharma and Singh 2024). The former distorts text within images and requires users to identify the characters, while the latter presents modified speech signals, prompting users to recognize spoken words (Snyder et al. 2010). These approaches rely on optical character recognition and automatic speech recognition technologies, respectively, to distinguish human users from automated bots (Wang et al 2023a; Pai et al. 2023).

Image-based CAPTCHAs have emerged as a robust solution to address the limitations of traditional security methods (Yamamoto et al. 2010). These typically involve visual tasks that leverage the human ability to identify objects and patterns-skills that remain comparatively difficult for computers to replicate accurately (Karpathy and Fei-Fei 2015). Despite advances in machine perception, image CAPTCHAs continue to present a significant challenge to automated systems. Nevertheless, further investigation into deep learning techniques is essential to understand how effectively such systems can detect and bypass these defenses (Mocanu et al. 2022).

Modern CAPTCHA systems face a growing array of challenges (Tariq et al. 2023). Chief among them is the need to design CAPTCHAs that can dynamically adapt to evolving attack strategies while remaining effective against emerging threats (Dinh and Hoang 2023). These systems must strike a careful balance-posing tasks complex enough to deter bots, yet remaining intuitive and user-friendly for legitimate users. Achieving this equilibrium is difficult, as overly sophisticated CAPTCHAs may frustrate users and reduce engagement (Wang et al. 2021). Moreover, with recent advances in deep learning, attackers can exploit powerful models for image recognition and reasoning tasks. Therefore, ensuring the resilience of CAPTCHA systems against such intelligent adversaries is essential for maintaining their security effectiveness (Magdy et al. 2021).

CAPTCHAs should be effective across various domains and scenarios, including text-based, image-based, and reasoning-based challenges. Designing CAPTCHA systems that generalize well across diverse contexts while maintaining both security and usability presents a significant challenge (Trong et al. 2023). Overcoming these challenges requires collaboration among researchers, developers, and industry stakeholders to drive innovation and continuously enhance CAPTCHA technologies in response to evolving threats and user needs.

**Fig. 1:**
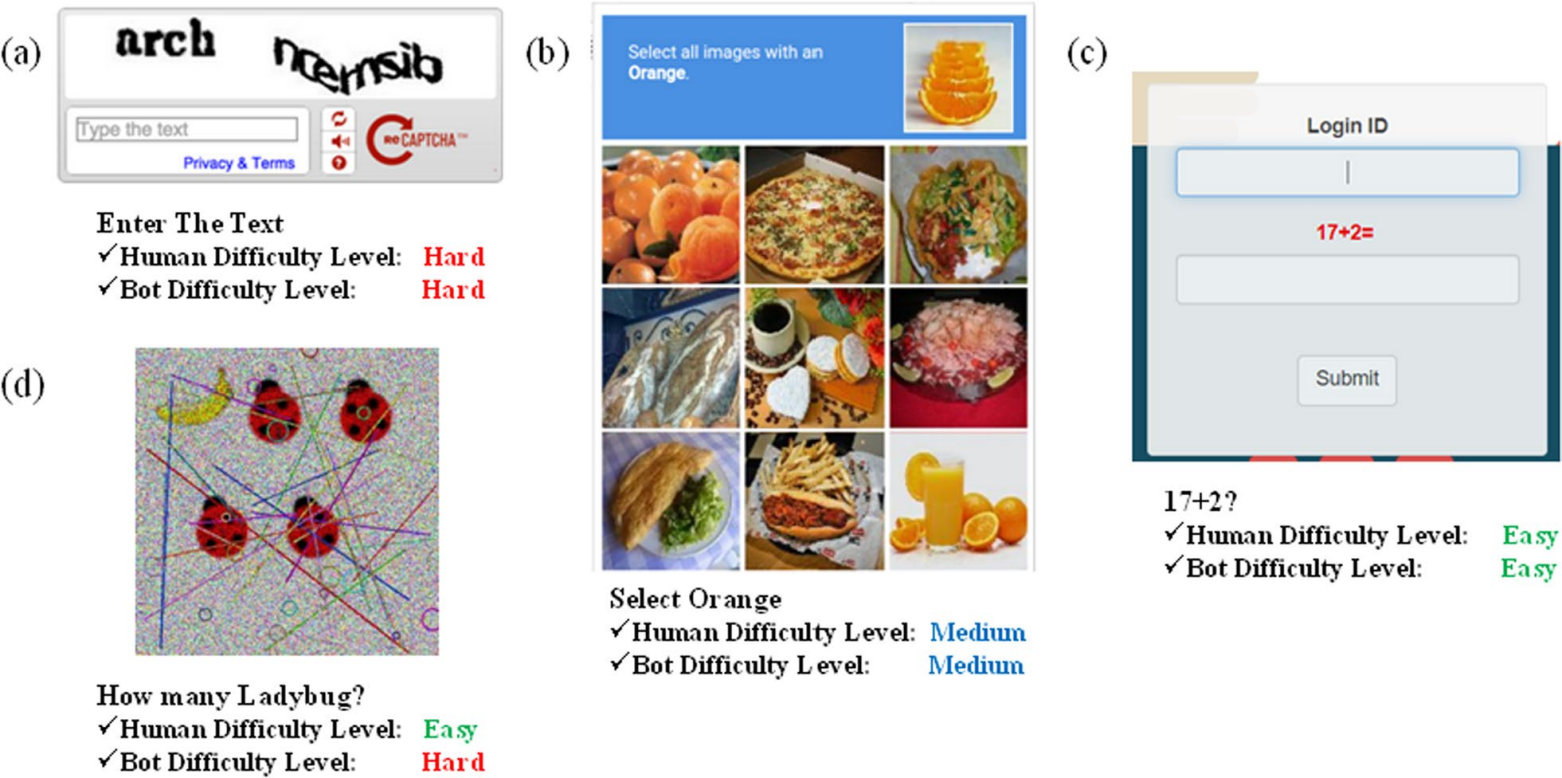
The state-of-the-art CAPTCHA systems: (**a**) Text-based image CAPTCHA, (**b**) Object-based image CAPTCHA, (**c**) Mathematical-logic-based image CAPTCHA, and (**d**) Proposed IReCAPTCHA

Fig. 1 illustrates state-of-the-art image and logic-based CAPTCHAs: (a) shows a text-based image CAPTCHA, where random words are deformed to create the CAPTCHA (Chen et al. 2017). If the deformation is too simple, it becomes easy for both humans and bots to solve. Therefore, the deformation must be complex to generate an effective CAPTCHA. As a result, this CAPTCHA is difficult for both bots and humans; (b) presents an object-based image CAPTCHA, which displays a set of random images and asks users to select matching objects for a given query object. Despite this approach, deep learning-based bots have shown considerable success in solving such challenges (Hossen et al. 2020); and (c) shows a mathematical-logic-based image CAPTCHA, where users solve a simple mathematical problem, such as addition or subtraction. However, recent advancements in solving mathematical word problems suggest that deep neural networks are increasingly capable of tackling these tasks effectively (Guerar et al. 2021).

This work proposes a new type of CAPTCHA that combines images and simple mathematical logic to enhance security. Our proposed CAPTCHA, shown in Fig. 1(d), incorporates both image deformation and mathematical logic, making it challenging for bots yet straightforward for humans to solve. To evaluate the robustness of our CAPTCHA design, we applied deep learning algorithms during testing. The key contributions of the proposed IReCAPTCHA are multifaceted:

- Combines image generation, noise modeling, and mathematical reasoning to create a more secure defense against automated attacks than traditional CAPTCHAs.
- Prioritizes user satisfaction by designing human-friendly questions that minimize friction during verification.
- Introduces dynamic image generation and mathematical reasoning, offering a novel method for mitigating automated threats while preserving accessibility.
- Provides a synthetic dataset of 5,000 CAPTCHA images, each paired with corresponding questions and answers, contributing a valuable benchmark for CAPTCHA-related research and algorithm evaluation.

The remainder of this article is organized as follows: Section 2 reviews and analyzes existing real-world CAPTCHAs. Section 3 describes the proposed IReCAPTCHA system. Section 4 presents the dataset, evaluation metrics, and experimental settings. Section 5 discusses the results and evaluates the effectiveness of the proposed image-based CAPTCHAs. Finally, Section 6 concludes the paper.

## Related Work

Various types of CAPTCHAs have been proposed in the literature to protect online services (Zhang et al. 2019; Xu et al. 2020; Johri et al. 2022). This section reviews related work from the past two decades, categorizing prior approaches into four subsections: noise-based, image-based, reasoning-based, and advanced AI-based CAPTCHAs.

## Noise-based CAPTCHA

### Image-based CAPTCHA

Noise is used in CAPTCHAs to make them challenging for bots to solve (Lorenzi et al. 2015b). Typically, this involves displaying a distorted image and asking users to recognize letters or numbers. Noise can be introduced in various ways, such as adding lines, circles, or dots to the image, blurring the image, or altering its contrast (Datta et al. 2009). Since line noise is commonly used on commercial websites, (Nakaguro et al. 2013) incorporated both line and salt-and-pepper noise in their CAPTCHA designs, achieving a precision of 0.83, recall of 0.86, and an F1-score of 0.89 in their experiments. Noise-based CAPTCHAs are difficult for bots, as bots lack the human capability to filter out visual noise. However, excessive noise can also make CAPTCHAs more difficult for users, underscoring the delicate balance between security and user experience in CAPTCHA design (Lorenzi et al 2015a). This highlights the importance of finding a suitable noise level-challenging for bots yet manageable for humans.

### Reasoning-based CAPTCHA

Reasoning-based CAPTCHAs require users to utilize their reasoning skills to complete a task. Examples include answering questions about images, solving puzzles (Ali and Karim 2014), or performing mathematical operations (Hernandez-Castro and Ribagorda 2010). Traditional mathematical CAPTCHAs often use basic arithmetic operations, such as addition, subtraction, multiplication, or division (Shirali-Shahreza and Shirali-Shahreza 2007). Over time, researchers have introduced more complex mathematical tasks, such as integration and polynomial operations. These require higher mathematical proficiency, limiting their applicability (Hernandez-Castro and Ribagorda 2010).

To address this limitation, researchers developed a handwritten mathematical sketch CAPTCHA called $\mu$captcha, inspired by the drawing CAPTCHA described in (Leiva and Alvaro 2015). The $\mu$captcha does not require users to have advanced mathematical knowledge; instead, users sketch a mathematical formula using a mouse or touchscreen while contending with an interference line designed to challenge bots but remain manageable for humans.

While the security of basic mathematical CAPTCHAs remains vulnerable to OCR attacks (Hernandez-Castro and Ribagorda 2010), reasoning-based CAPTCHAs continue to evolve. They hold the potential for greater difficulty compared to traditional image-based CAPTCHAs. Table 1 summarizes various image-reasoning CAPTCHAs and their robustness levels against deep learning-based attacks.

**Table 1:** Image-reasoning CAPTCHAs and deep learning attacks Resistance

Image-Reasoning CAPTCHA	Example	Attack Requires	Deep-Learning Attack Resistance
Object Counting	Ask the user to count specific objects in an image (e.g., "How many cats are there?")	Requires basic object recognition	Low
Object Comparison	Ask the user to compare objects in an image (e.g., "Which object is bigger, the tree or the house?")	Requires basic object recognition and relative size comparison	Moderate
Scene Understanding	Ask the user to answer questions about the scene depicted (e.g., "What is happening in the image?")	Requires higher-level reasoning and scene understanding.	High
Dynamic Manipulation	Present images with dynamic elements or require user interactions (e.g., click on a specific object after it moves).	Leverages random information, making it difficult for static deep-learning models	Very High
Multi-Modal Integration (Proposed)	Combines random images with other modalities, such as adding random noise and random questions for reasoning (e.g., "Count a specific object in a randomly generated noisy image")	Requires diverse information for noise processing to recognize objects and language understanding for random questions	Extreme High

### Advanced AI-based CAPTCHA

AI-based CAPTCHA solvers have significantly advanced, particularly with the rise of capsule networks, which enhance image recognition capabilities in CAPTCHA-breaking algorithms (Mocanu et al. 2022). To counter these developments, recent adversarial CAPTCHA defenses employ adaptive, noise-based techniques that adjust to specific AI threats. For example, capsule networks can be disrupted using randomized noise patterns, making CAPTCHAs more challenging for AI-based attacks (Moradi et al. 2024). Furthermore, adaptive CAPTCHA systems now incorporate real-time analysis of user input, dynamically adjusting complexity to maintain a balance between accessibility and security. These adaptive approaches have shown promising improvements in user experience without compromising security (Dinh et al. 2023).

## Proposed Model

**Fig. 2:**
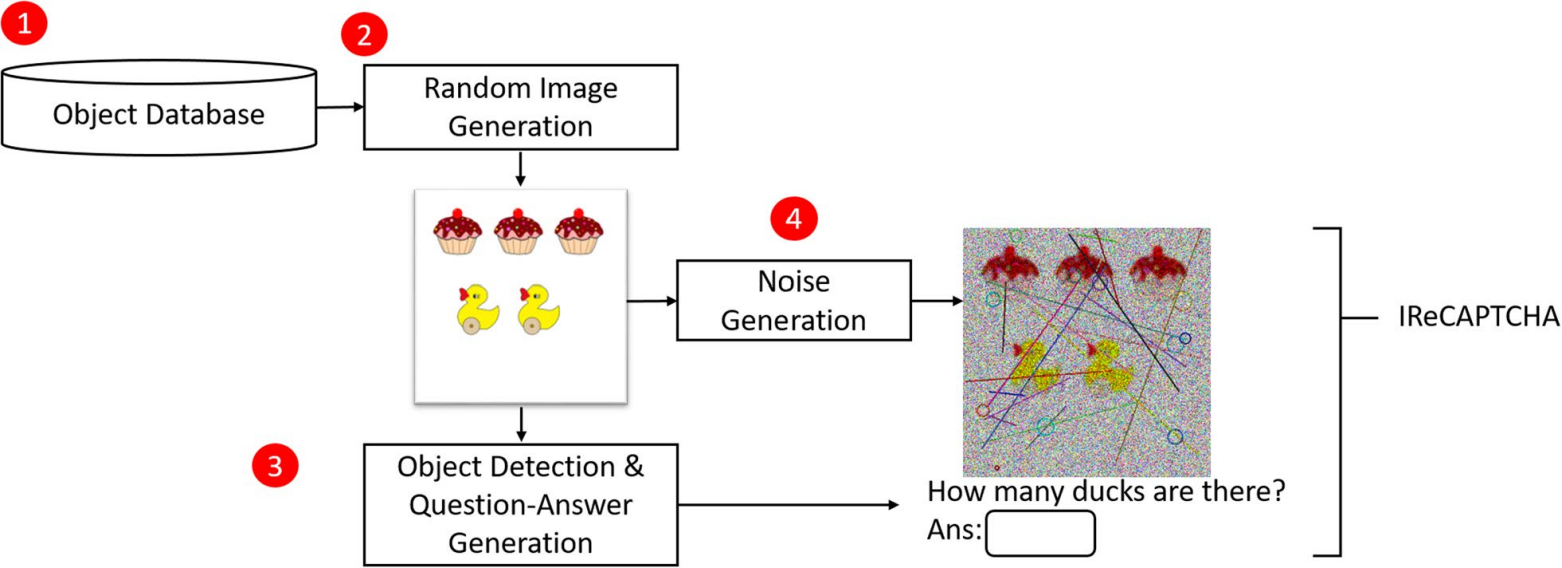
The proposed IReCAPTCHA system, including the image generation module, noise generation module, and question-answer generation module

The proposed IReCAPTCHA framework comprises three core components: (1) a random image generation module, (2) an object detection module for question–answer pair generation, and (3) a noise generation module. Fig. 2 presents the complete architectural overview of the proposed system.

**Fig. 3:**
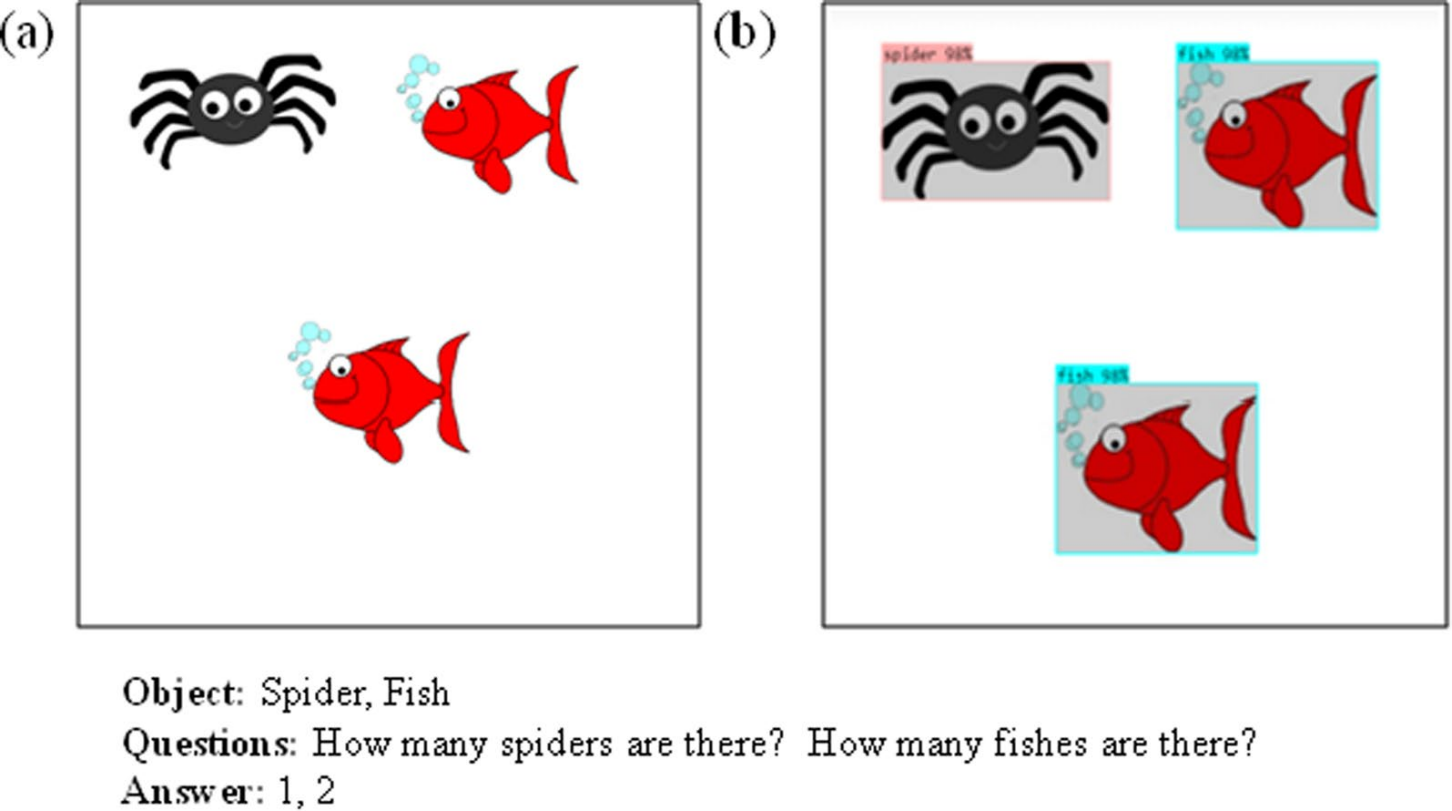
The question-answer generation from a random image using object detection (Das et al. 2021), (a) Input image, and (b) Object detection bounding boxes

The object database, which contains various objects and shapes, is regularly updated to enhance the proposed CAPTCHA system. Initially, the model generates multiple images from this object database. It then utilizes image understanding capabilities to formulate relevant questions and corresponding answers for each image. Figure 3 illustrates this image-based question–answer generation process.

**Fig. 4:**
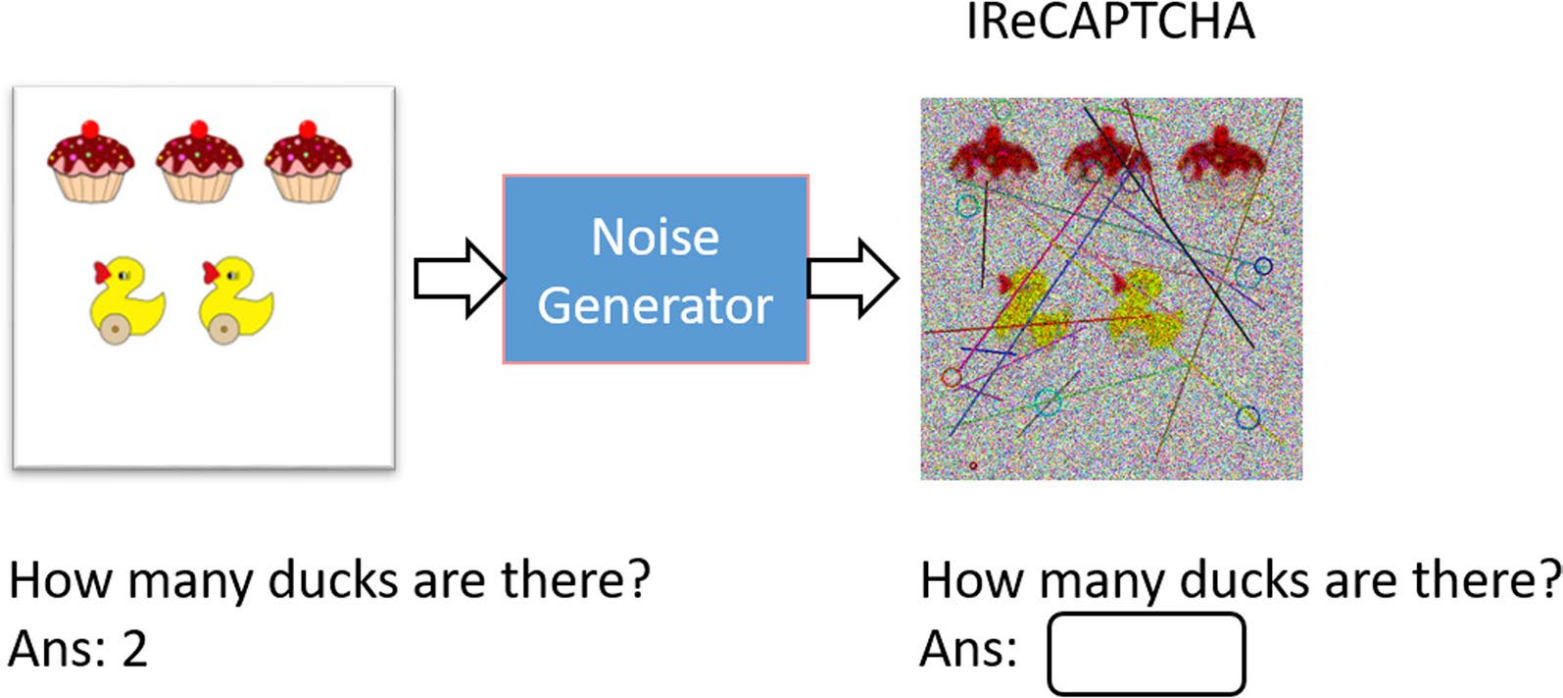
The IReCAPTCHA generation process: (**a**) Input image and (**b**) Final CAPTCHA image after applying noise

Subsequently, a noise generator introduces carefully crafted noise patterns to a randomly selected image, producing the final IReCAPTCHA. The output of this process-demonstrating CAPTCHA generation after noise addition-is shown in Figure 4. The following sections provide detailed descriptions of each module.

### Question and answer (Q&A) generation

The object dataset *O* contains diverse objects and shapes, each with multiple color variations. Formally, we define the dataset as:

1 $$O = \{o_1, o_2, \ldots , o_N\}$$

where $o_i$ represents a distinct object in the dataset. To enhance CAPTCHA robustness, we implement a dynamic update mechanism that periodically refreshes the object collection and automatically harvests new elements from web sources at fixed intervals.

For image generation, each composite image *I* combines one to three randomly selected objects from *O*, as follows:

2 $$\begin{aligned} I = \{o_j, o_k, o_l\}, \quad {\mathrm{where}}\; o_j, o_k, o_l \in O\; {\text { and }}\; 1 \le |I| \le 3 \end{aligned}$$

The system maintains a knowledge base $I_{\mathrm{knowledge}}$, which tracks object frequencies:

3 $$\begin{aligned} I_{\mathrm{knowledge}} = \{\langle o_1, c_1 \rangle , \langle o_2, c_2 \rangle , \ldots , \langle o_M, c_M \rangle \} \end{aligned}$$

where $c_i$ denotes the count of object $o_i$. From this, we generate question-answer pairs:

4 $$\begin{aligned} I_{{{\mathrm{question}}}} & = \{ ``{\text{How many}}\;o_{i} \;{\text{are there}}\;?{\mathrm{''}}{\mid }\langle o_{i} ,c_{i} \rangle \in I_{{{\mathrm{knowledge}}}} \} \\ {\mathrm{Answer}} & = c_{i} \\ \end{aligned}$$

Our implementation pipeline consists of: (1) random image generation with object and shape variations, (2) multi-description extraction using the VGG-16 convolutional neural network (CNN), and (3) sequence modeling via long short-term memory (LSTM) networks for question–answer (Q&A) generation. The image-based question–answer generation methodology follows the approach described in Das et al. (2021), with enhancements tailored to our CAPTCHA application.

### Noise model

The proposed noise model employs multiple complementary distortion techniques to generate CAPTCHAs that balance security and human solvability. Specifically, we integrate five noise types in varying combinations: (1) Gaussian noise, which introduces additive random perturbations; (2) salt-and-pepper noise, which causes random pixel corruption; (3) line noise, used for strategic obstructions; (4) circular noise, forming concentric interference patterns; and (5) speckle noise, which applies multiplicative random variations.

$$Noise{\mkern 1mu} Composition{\mkern 1mu} N(I) = \underbrace {{g_{\sigma } }}_{{Gaussian}}^\circ \underbrace {{S_{p} }}_{{S[NONSPACE]\& p{\text{ }}}}^\circ \underbrace {{sp_{\alpha } }}_{{Speckle}}^\circ \underbrace {{l_{n} }}_{{Lines}}^\circ \underbrace {{c_{r} }}_{{Circles}}(I){\text{ }}$$

**Fig. 5:**
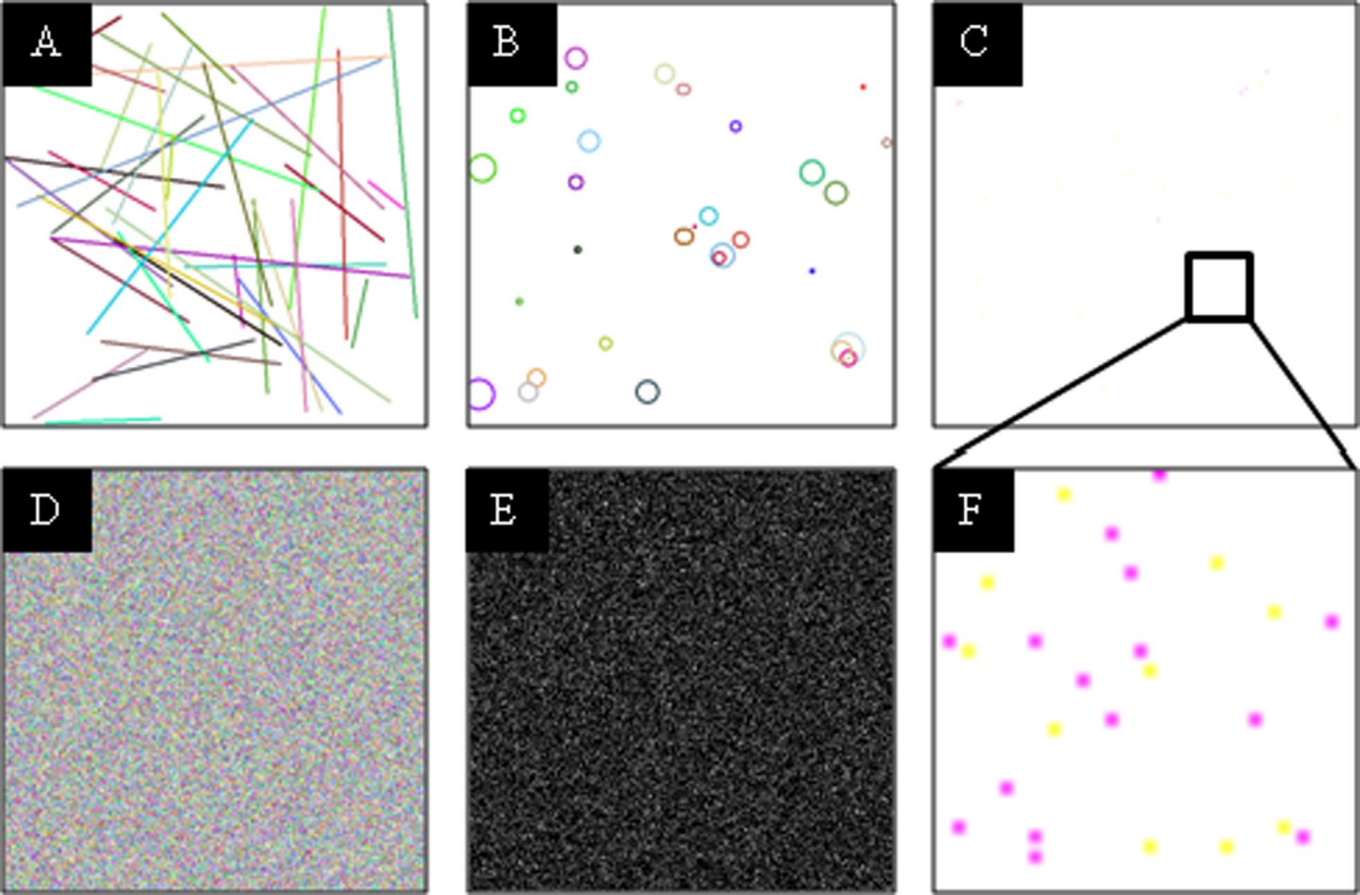
The different noises applied in the proposed noise model: (A) Line noise, (B) Circular noise, (C) Salt-and-pepper noise (zoomed-in shown in F), (D) Speckle noise, and (E) Gaussian noise in an inverted form

This composite noise approach produces images that are computationally challenging for automated systems while remaining interpretable to human users. The visual characteristics of each noise type are illustrated in Figure 5, and the complete noise application procedure is formally described in Algorithm 1.

**Algorithm 1:**
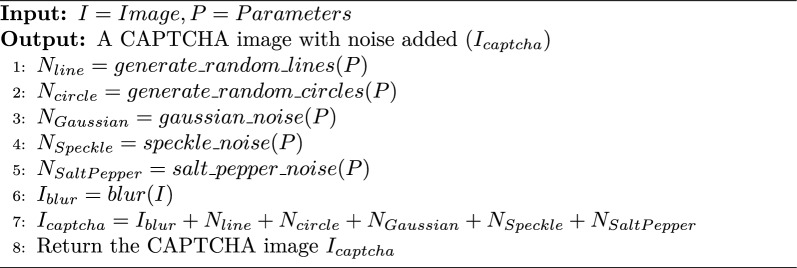
NOISE Model

**Fig. 6:**
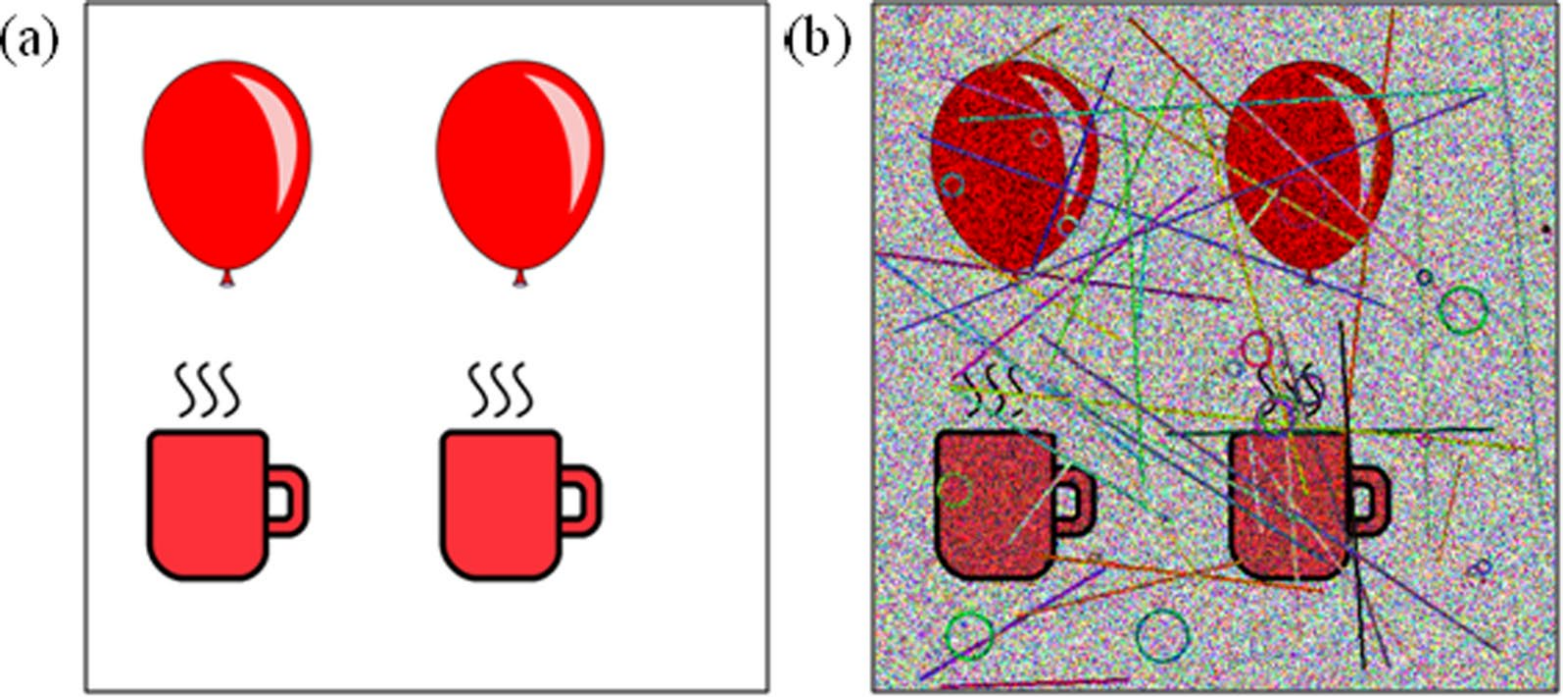
I$_{captcha}$ generation: (a) Input image, and (b) Final image with noises

While the described noise model represents an effective approach for CAPTCHA generation, numerous alternative noise-adding techniques exist in the literature. The optimal noise configuration depends fundamentally on two key factors: (1) the desired security level (i.e., human solve-rate versus bot resistance), and (2) the characteristics of the base images. Figure 6 illustrates this process through a visual comparison, showing both the original input image and its transformed counterpart after noise application.

### CAPTCHA generator

The CAPTCHA generator module takes input from the preceding components to produce a CAPTCHA. The process involves selecting random objects from the dataset and applying multiple layers of noise-such as Gaussian, salt-and-pepper, speckle, line, and circular noise-which significantly challenge AI recognition systems. Once the noisy image is generated, the algorithm uses an object detection module (VGG-16 CNN) to identify objects within the image. It then employs a LSTM network to formulate a question based on the detected objects, such as counting or identifying specific items. The final output, IReCAPTCHA, consists of the noisy CAPTCHA image and the generated question, creating a CAPTCHA that requires both visual comprehension and logical reasoning. Algorithm 2 outlines the steps of CAPTCHA generation.

**Algorithm 2:**
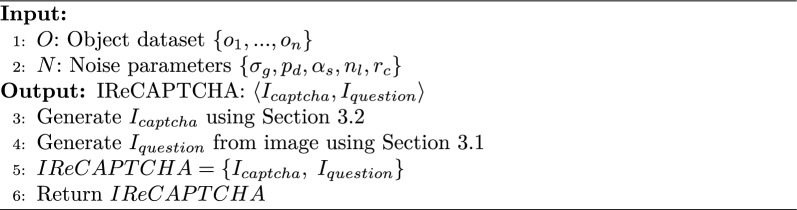
CAPTCHA Generator

The algorithm leverages both advanced noise application and dynamic question generation to produce CAPTCHA images that are accessible to humans but obstructive to AI systems. This method enhances security by emphasizing image interpretation and logical reasoning, moving beyond traditional object detection and text-based CAPTCHA approaches.

### IReCAPTCHA for visually impaired users

To enhance accessibility for visually impaired (VI) users, the IReCAPTCHA system is adapted into a simple mathematical word problem (MWP). This approach prioritizes clarity and simplicity, allowing VI users to solve problems without requiring advanced mathematical skills-thus balancing security and accessibility. Instead of presenting a noisy image, the system generates an audio or text-based question derived from object-based CAPTCHA logic. Algorithm 3 outlines the process for generating CAPTCHAs tailored to visually impaired users. The following sets and parameters are utilized in the generation process:

- The set of objects in the CAPTCHA, denoted by $O_{\text {object}} = \{o_1, o_2, \dots , o_M\}$
- The count of objects, represented as $O_{\text {count}} = \{c_1, c_2, \dots , c_M\}$
- The set of operations, $OP = \{\text {add, subtract}\}$
- The set of numbers, $N = \{0, 1, \dots , 10\}$

**Algorithm 3:**
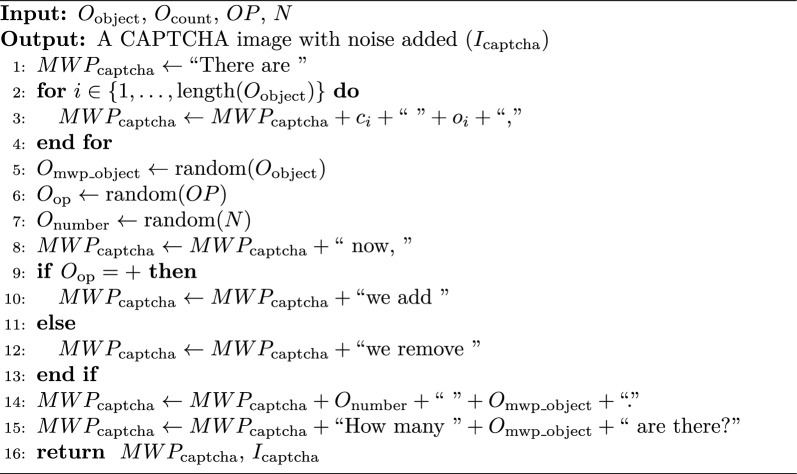
CAPTCHA for Visually Impaired Users

**Fig. 7:**
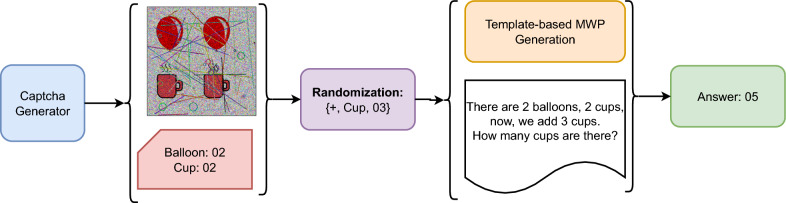
The flow illustrating the proposed $MWP_{captcha}$ generation process for visually impaired users

To enhance accessibility for visually impaired (VI) users, the IReCAPTCHA system is adapted into a simple mathematical word problem (MWP). This approach prioritizes clarity and simplicity, allowing VI users to solve problems without requiring advanced mathematical skills-thus balancing security and accessibility. Instead of presenting a noisy image, the system generates an audio or text-based question derived from object-based CAPTCHA logic (Fig. 7). Algorithm 3 outlines the process for generating CAPTCHAs tailored to visually impaired users. The following sets and parameters are utilized in the generation process:

## Experiment

### Dataset

We generated a dataset of 5,000 synthetic image CAPTCHAs using a simulator, along with their associated questions and corresponding answers. This dataset was used to train a solver capable of identifying objects within the images and answering the corresponding CAPTCHA questions. Our CAPTCHA solver is based on the YOLOv8 object detection model. The trained solver takes a CAPTCHA image and a question as input and generates the corresponding answer. We will publish our dataset at https://github.com/bidyut2002in/CAPTCHA.

### Experimental Setup

We conducted our experiments in Python using standard open-source libraries (e.g., OpenCV) and implemented the YOLOv8 object detection model (Jocher et al 2023), a state-of-the-art framework for real-time detection. The model was trained using a PyTorch-based implementation of YOLO, incorporating learning rate scheduling with decay and early stopping to prevent overfitting. The dataset was split 70–30 for training and testing, and the entire test set was subjected to various noise levels to evaluate robustness.

### Performance Measures

**Fig. 8:**
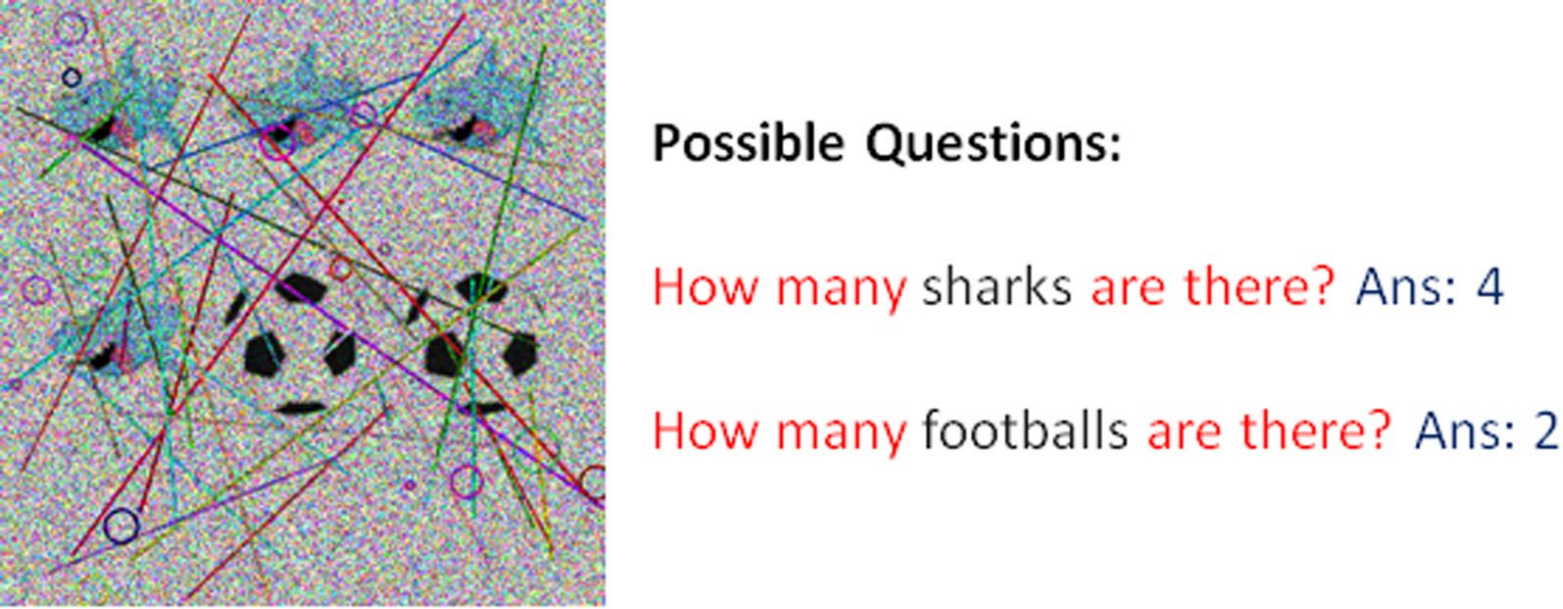
Example of the proposed IReCAPTCHA showing the combination of noisy CAPTCHA images and corresponding questions

Our proposed CAPTCHA system challenges users to verify their humanity by solving a task that combines randomly generated images with questions selected from a predefined set (Fig. 8). These questions, designed to be intuitive for humans, require identifying objects within the image. The system’s primary technical challenge lies in achieving robust object detection, as the accuracy of this step directly determines the solver’s ability to respond correctly.

To evaluate performance, we focus exclusively on object detection accuracy, since the CAPTCHA’s validity hinges entirely on this metric. The proposed method was assessed using the following two key performance measures. All experiments were conducted on an NVIDIA A5000 GPU to ensure computational efficiency.

**Precision:** It is the ratio of true positives (*TP*) to the total number of positive predictions $(TP + FP)$. Alternatively, precision quantifies the proportion of predicted positives that are truly relevant. It is defined as

5 $$\begin{aligned} Precision = \frac{TP}{TP+FP} \end{aligned}$$

**Recall:** It is the ratio of true positives (*TP*) to the total number of actual positive cases $(TP + FN)$. Alternatively, recall measures the proportion of actual positives that are correctly identified. It is defined as

6 $$\begin{aligned} Recall = \frac{TP}{TP+FN} \end{aligned}$$

## Results and discussion

The study investigates prominent deep learning-based approaches used to attack CAPTCHAs, encompassing character recognition (Noury and Rezaei 2020), object detection (Sivakorn et al 2016), and inverse problem-solving (Zou et al. 2021). For example, Noury and Rezaei (2020) introduced a technique leveraging deep learning to identify and extract textual elements from noisy images-an enduring challenge for CAPTCHA solvers. Likewise, Sivakorn et al (2016) employed YOLOv3, a robust object detection model, to address CAPTCHAs that contain real-world objects. Although these strategies demonstrate efficacy on image-text-based CAPTCHAs, they tend to neglect tasks involving mathematical reasoning.

To evaluate CAPTCHA security more robustly, this study employs YOLOv8 (Wang et al. 2023b), a state-of-the-art object detection model. We trained the detection module using a novel CAPTCHA dataset and evaluated its performance under various noise conditions. Remarkably, the proposed noise model drastically reduces the efficacy of object detection-based attacks-even when leveraging an advanced framework such as YOLOv8. While YOLOv8 achieves high accuracy on conventional CAPTCHA formats, its detection rate plummets by up to 99% when tested against our noise-enhanced CAPTCHA variants.

**Fig. 9:**
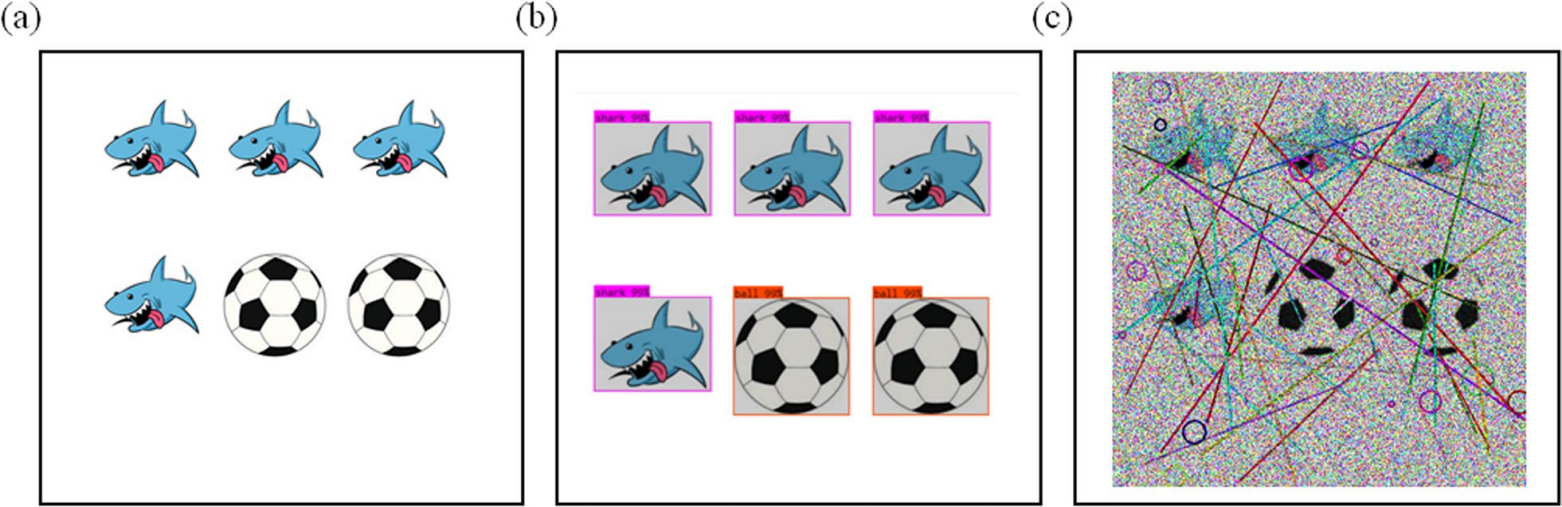
Comparison of CAPTCHA resistance to object detection-based attacks: (**a**) Standard CAPTCHA without noise, (**b**) Successful detection of objects in noise-free CAPTCHA, and (**c**) Proposed noisy IReCAPTCHA showing failure of object detection models

Fig. 9 illustrates the strength of our approach, contrasting successful detections in noise-free CAPTCHAs with failures in the noise-infused IReCAPTCHA. These findings underline the resilience of the proposed method against contemporary deep learning-based attacks and underscore the critical need for integrating multi-layered noise defenses into CAPTCHA design.

The following subsections provide a comprehensive evaluation of IReCAPTCHA. We begin with an ablation study to examine the contribution of each system component. Next, we present a comparative analysis against existing CAPTCHA techniques, followed by an investigation of system robustness under increasing noise levels. Subsequently, we assess the time complexity of CAPTCHA generation and explore the impact of various denoising strategies. A detailed failure analysis is also included, culminating in the application of our approach to more complex, real-world visual datasets.

### Ablation study

In the ablation study, we systematically assessed the impact of each component of the proposed system by removing them individually. This methodology enabled us to quantify the contribution of each element to the overall system performance. By examining variations in the F1-score across different configurations, we gained valuable insights into how the absence of specific components influenced object detection accuracy. This rigorous evaluation highlighted the components critical for achieving optimal performance. The corresponding results are summarized in Table 2.

**Table 2:** Ablation study of the proposed IReCAPTCHA’s performance using an object detection-based CAPTCHA solver

**Input to the solver by degrading image**	**Precision**	**Recall**	**F1 Score**
No degradation	0.95	0.92	0.93
Blur	0.88	0.85	0.86
Line Noise	0.80	0.78	0.79
Speckle Noise	0.82	0.79	0.80
Salt-and-Pepper Noise	0.75	0.72	0.73
Blur + Line + Speckle	0.70	0.68	0.69
All Noise + Reasoning (IReCAPTCHA)	0.035	0.030	0.032

### Comparison with existing CAPTCHA methods

Table 3 presents a comparative evaluation of IReCAPTCHA against existing CAPTCHA systems under varying conditions. The proposed method consistently surpasses traditional approaches, including YOLOv3-based solvers and $\mu$CAPTCHA, especially in environments with minimal or no noise. Notably, IReCAPTCHA demonstrates strong resilience to complex noise patterns, whereas competing methods suffer substantial accuracy degradation. While the integration of reasoning tasks markedly improves security, performance under compounded noise conditions indicates room for further refinement to bolster robustness against sophisticated automated attacks.

**Table 3:** Comparative analysis of the proposed IReCAPTCHA against existing CAPTCHA systems

**Method/Study**	**Noise Level**	**Precision, Recall, F1-Score**	**Comments**
YOLOv3 (Sivakorn et al 2016)	No Noise	0.98, 0.96, 0.97	High performance for simple text CAPTCHA; struggles with advanced noise challenges.
DeepCAPTCHA (Noury and Rezaei 2020)	Moderate Noise	0.85, 0.82, 0.83	Deep learning for image CAPTCHAs; limited with complex noise/reasoning.
$\mu$CAPTCHA (Leiva and Alvaro 2015)	No Noise	0.87, 0.85, 0.86	User-drawn input without reasoning or noise mitigation.
Adversarial Defense (Moradi et al. 2024)	Adaptive Noise	0.83, 0.79, 0.81	Adaptive techniques lack reasoning integration.
**IReCAPTCHA (Proposed)**	**Combined Noise**	**0.035, 0.030, 0.032**	**Excels in complex noise with integrated reasoning.**

### Effect of noise on GPT-4o and human solvers

Recent advancements in large visual models (LVMs), including GPT (Achiam et al. 2023), LLaVA (Li et al. 2024), and Gemini (Team et al. 2023), have introduced new challenges to CAPTCHA design. In this study, we assess the performance of an automated CAPTCHA solver utilizing GPT-4o and compare it with human accuracy.

**Fig. 10:**
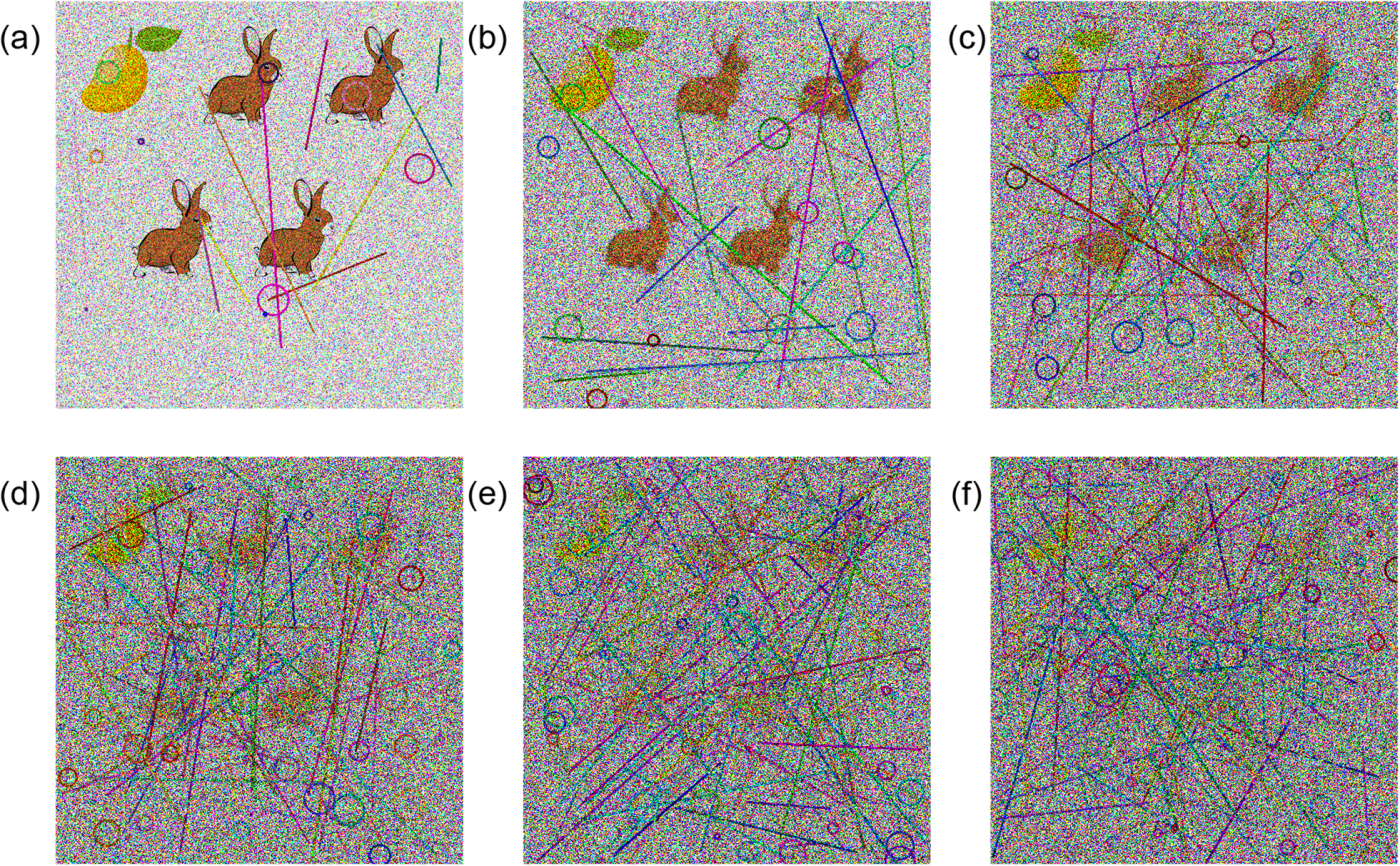
Examples of different noise levels. (**a**) 1, (**b**) 6, (**c**) 12, (**d**) 18, (**e**) 24, and (**f**) 30

**Fig. 11:**
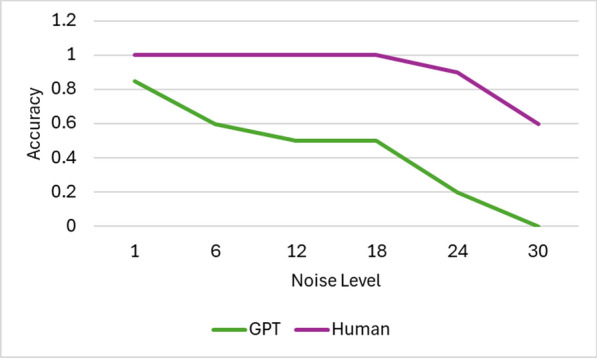
Performance of GPT-4o and humans varying different noise levels

We conducted evaluations across 30 distinct noise levels, beginning with the following initial parameters: $\text {blur\_level} = 1$, $\text {number\_of\_lines} = 10$, $\text {number\_of\_circles} = 10$, and $\text {noise\_level} = 1$. For each successive level, the blur intensity was increased by 1, the number of lines and circles by 5, and the intensity of salt-and-pepper, Gaussian, and speckle noise was incremented by 0.1. Figure 10 illustrates these noise variations. The comparative performance of GPT-4o and human participants is depicted in Figure 11.

As noise levels increase, the performance of both GPT and human users exhibits a declining trend. Notably, noise level 24 appears to strike a favorable balance-human users maintain high accuracy while GPT-based solvers struggle considerably. Table 4 presents comparative human usability metrics across different noise levels. Furthermore, performance fluctuations are observed based on the dataset type, indicating that dataset characteristics significantly influence solver robustness.

**Table 4:** Comparative human usability metrics under different noise levels. Noise level 24 represents the optimal balance between security and user experience

**Noise Level**	**Avg. Solve Time (sec)**	**Success Rate (%)**	**User Feedback (1–5)**	**Feedback Summary**
0 (No Noise)	4.2	99.2	4.8	Very easy to solve.
6	5.1	97.8	4.5	Slightly challenging but manageable.
12	6.3	95.4	4.1	Takes a bit more time, but fair.
18	8.0	91.0	3.7	Challenging but understandable.
**24 (Optimal)**	**8.8**	**89.1**	**4.2**	Good balance of difficulty and usability.
30	11.6	75.5	2.8	Too noisy, frustrating to interpret.

### Time complexity analysis

The time complexity of the IReCAPTCHA generation process can be modeled based on three primary operations: object selection, noise addition, and template-based question generation. Assuming a database of *n* objects, selecting and placing *m* randomly chosen objects into the image template takes *O*(*m*) time. Next, *k* distinct noise types are applied, each affecting up to *p* pixels in the image. This results in a cumulative noise complexity of $O(k \cdot p)$. Finally, the template-based question-and-answer generation-relying primarily on simple rule-based logic or object frequency reasoning-adds an additional *O*(*m*) complexity. Combining these operations, the overall time complexity for generating a single IReCAPTCHA can be expressed as $O(m + k \cdot p)$, where the cost scales linearly with the number of visual elements and the intensity of noise applied.

**Fig. 12:**
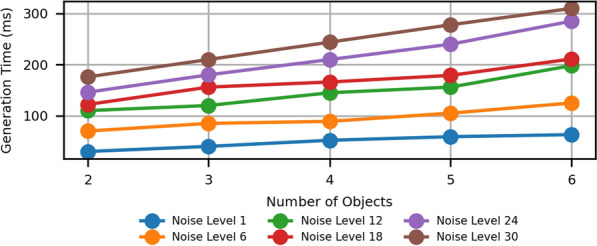
Average IReCAPTCHA generation time, varying the number of objects and noise level.

We conducted experiments on a real-world setup using an Intel i7 CPU to generate CAPTCHAs. We tested varying numbers of objects and noise levels. We repeated the experiment 100 times for each case, and the average time is reported in Figure 12.

### Impact of denoising techniques

One of the key contributions of this study is the design of a noise model that poses significant challenges for interpretation by both conventional machine learning and modern deep learning algorithms. We evaluate the effectiveness of various denoising techniques on automated CAPTCHA solvers, including the Diffusion V5 model (?), NAFNet pretrained on the SIDD dataset (?), mean denoising (?), and GPT-4o pretrained models. For Diffusion V5 and GPT-4o, we applied the prompt “denoise image and preserve the original color and texture”.

**Fig. 13:**
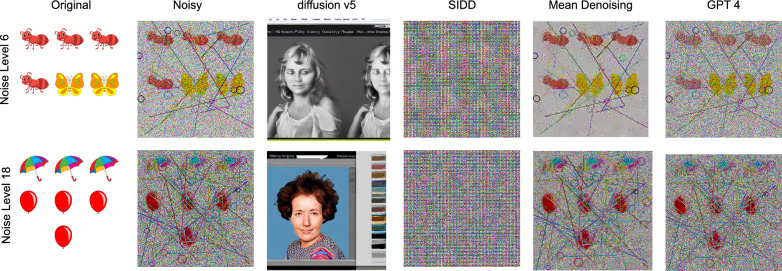
Denoising IReCAPTCHA using different methods

Figure 13 presents the denoising outcomes for two representative examples from noise levels 6 and 18. The Diffusion-based method generates visually inconsistent outputs, likely due to its training on heterogeneous real-world image datasets. We believe that retraining the model can improve the denoising task, but this is out of the scope here. NAFNet also fails to remove noise effectively, attributed to the mismatch in its pretrained model. Mean denoising yields better results than GPT-4o; however, both methods exhibit diminished performance at higher noise levels. Notably, object detection-based CAPTCHA solvers demonstrate only a marginal F1-score improvement of 0.004 after denoising, while GPT-based solvers show an even lower gain of 0.002.

### Error and failure analysis

Table 5 summarizes the observed failure cases in which GPT-4o generated incorrect responses. Notable error patterns include overcounting-such as hallucinating non-existent objects-color misidentification under Gaussian noise, and misclassification induced by circular distortions. These findings reinforce the critical role of multi-layered noise in impairing the perceptual and reasoning capabilities of automated solvers.

**Table 5:** Error analysis comparing GPT-4o answers to ground truth on noisy CAPTCHAs

Image	Question	Ground-Truth Answer	GPT-4o Answer	Error Reason
	How many blue circles are there?	5	7	Two extra circles were mistakenly counted due to noise.
	How many teapots are there?	5	6	GPT hallucinated one extra teapot in a blurred background area.
	How many green triangles are there?	9	7	Failed to distinguish color differences under Gaussian distortion.
	How many stars are there?	2	6	Misclassified shapes under heavy circular noise interference.
	How many cupcakes are there?	9	7	Too noisy, GPT unable to detect the object properly.

### Handling advanced real-world datasets

**Fig. 14:**
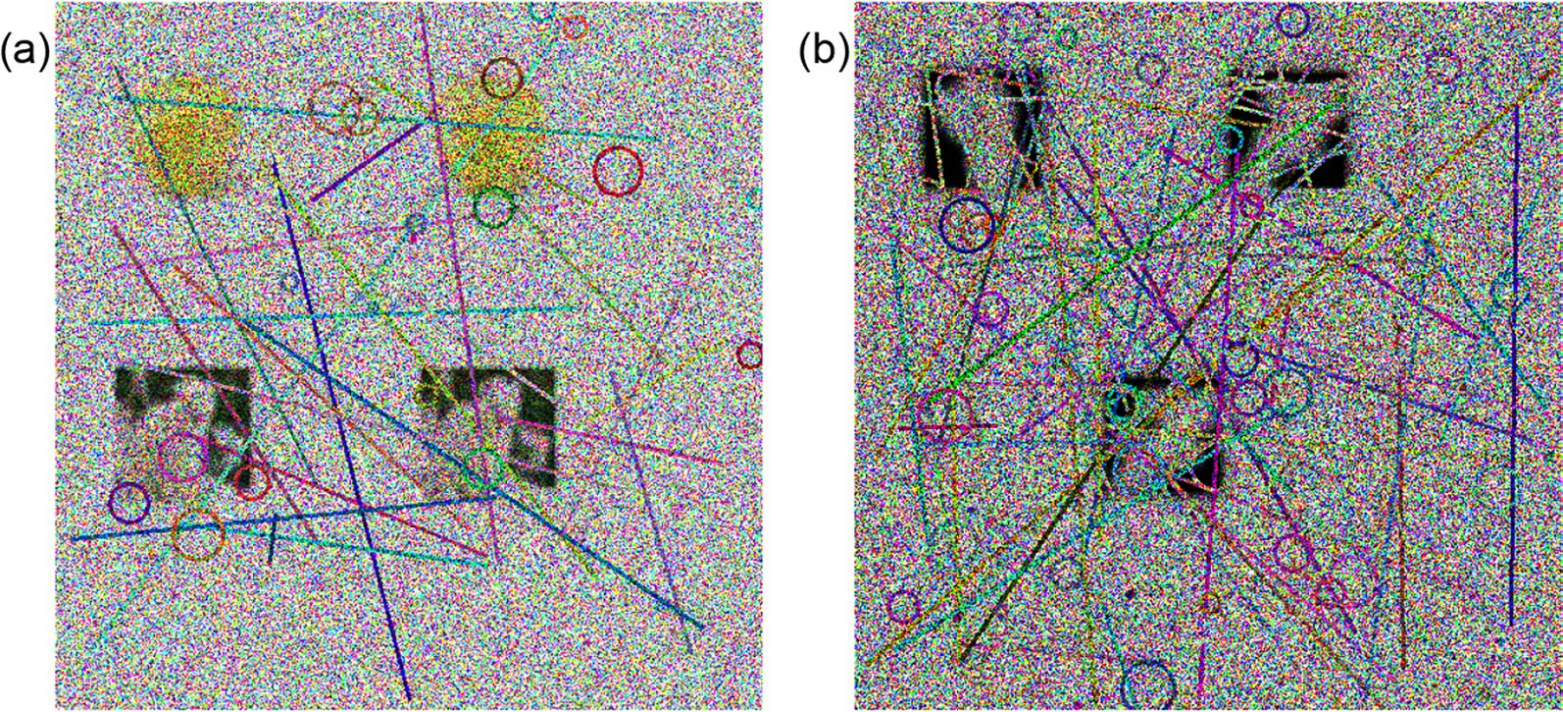
Examples of different CAPTCHA. (**a**) Generated from CIFAR-100 with noise level 6 Q: How many beers are there?, (**b**) Generated from MNIST with noise level 20 (Q: How many sevens are there?

IReCAPTCHA introduces mechanisms to escalate CAPTCHA complexity through the integration of real-world objects. By leveraging datasets such as CIFAR-100 and MNIST, we can generate CAPTCHA instances with tunable noise levels, thereby intensifying the challenge for automated solvers. Fig.  14 illustrates two representative examples, demonstrating the system’s ability to synthesize diverse and perceptually demanding CAPTCHA tasks grounded in widely used visual datasets.

**Fig. 15:**
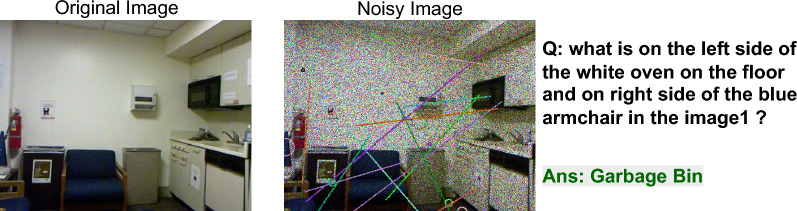
Examples of noisy CAPTCHA generated from a VQA dataset

Image-based visual question answering (VQA) datasets can also be integrated into the CAPTCHA framework. By injecting the proposed noise into real-world images from VQA datasets and posing complex questions, the system generates CAPTCHAs that challenge automated solvers while remaining solvable by humans. For instance, Fig. 15 illustrates an example taken from a VQA dataset ?, where the applied noise significantly impairs solver performance but still permits human interpretation.

## Conclusion

This paper presents IReCAPTCHA, a novel CAPTCHA framework designed to enhance security against automated bot attacks. By integrating advanced image understanding, noise mitigation, and mathematical reasoning, IReCAPTCHA delivers superior protection compared to existing CAPTCHA systems. Despite its robust defense mechanisms, IReCAPTCHA maintains a user-friendly interface, offering tasks that remain intuitive for humans while challenging for machines.

Extensive evaluations against object detection-based attacks demonstrate the system’s effectiveness in preventing automated access, reinforcing its applicability in real-world scenarios. Future enhancements will focus on the development of 3D-image CAPTCHAs, leveraging spatial relationships among objects to further complicate automated recognition. Additionally, we aim to implement adaptive security mechanisms that dynamically adjust CAPTCHA complexity based on evolving threat vectors and user interactions. The incorporation of multi-modal elements-such as audio and visual cues-will further strengthen security while improving accessibility for diverse user populations.

These advancements position IReCAPTCHA as a resilient and forward-looking solution for securing websites and online services against increasingly sophisticated bot activity.

## Data Availability

The datasets generated and/or analysed during the current study are available in the GitHub repository, https://github.com/bidyut2002in/CAPTCHA.
